# Cognitive Emotion Regulation Questionnaire—Short: Reliability, Validity, and Measurement Invariance of the Italian Version

**DOI:** 10.3390/bs12120474

**Published:** 2022-11-24

**Authors:** Silvia Cerolini, Andrea Zagaria, Mariacarolina Vacca, Philip Spinhoven, Cristiano Violani, Caterina Lombardo

**Affiliations:** 1 Department of Psychology, Sapienza University of Rome, 00185 Rome, Italy; 2 Institute of Psychology, Leiden University, 2333 AK Leiden, The Netherlands

**Keywords:** cognitive emotion regulation, emotions, assessment, emotion regulation strategies, validation study

## Abstract

Objective: The Cognitive Emotion Regulation Questionnaire (CERQ) is a widely used instrument to assess cognitive emotion regulation strategies. The study aimed to test the psychometric properties of the Italian short version of the CERQ (CERQ-IS). Methods: Two separate samples of 442 young adults (M_age_ = 21.12; SD = 3.69) and 256 adolescents (M_age_ = 14.81; SD = 0.59) completed the CERQ, the Emotional Regulation Questionnaire (ERQ) and the Multidimensional Perfectionism Scale (MPS). A confirmatory factor analysis (CFA) was performed to evaluate the dimensionality of the CERQ-IS. Internal consistency was analysed by calculating model-based composite reliability coefficients. Criterion and discriminant validity were gathered through the correlations with the ERQ and the MPS, respectively. Factorial invariances tests across gender and age were computed by means of multiple-group CFA. Results: CFA confirmed the nine-factor structure showing an excellent fit to the data. Except for rumination which was minimally acceptable, all subscales had an acceptable to good reliability. Criterion validity was supported by significant correlations between CERQ-IS and ERQ subscales. Discriminant validity was confirmed by meaningless correlations with the MPS facets. Configural, metric and scalar invariance were established across both grouping variables. Conclusions: The brevity of this tool and its good psychometric properties suggest that CERQ-IS could be a useful screening tool in both clinical and research practice in adolescence and young adulthood.

## 1. Introduction

Emotional regulation refers to the ability to recognize and distinguish one’s emotions and the ability to manage the intensity and duration of the emotional experience [1]. These abilities involve the use of different cognitive and behavioral strategies which, if used flexibly, make the emotional experience enriching, functional, and balanced with respect to both positive and negative emotions [2,3].

The ability to adaptively regulate emotions is crucial for social functioning and mental health [1,4]. Some emotion regulation strategies are considered risk factors for psychopathology, while others are considered protective factors [5]. Consistently, difficulties in emotion regulation have been associated with psychopathological disorders and emotional problems such as anxiety and eating disorders [6,7], depression [8,9], insomnia [10,11], generalized anxiety disorder [12], and obsessive compulsive disorder [13]. Cognitive emotion regulation strategies such as self-blame, rumination, catastrophizing, and positive reappraisal (inversely) were found to be associated with negative emotional states like depression, anxiety, stress, and anger, e.g., [7,14,15,16,17]. Considering this theoretical framework, the regulation of emotions is of fundamental importance in some “turning point” moments in life, such as adolescence or the transition to university and adult life [18,19,20].

In order to assess emotion regulation abilities and strategies, various instruments have been developed and validated so far. One of them is the Cognitive Emotion Regulation Questionnaire (CERQ) [7], a 36-item scale, which was adapted into several languages, and its good psychometric properties demonstrated in France [17], China [21], Romania [22], Hungary [23], Iran [24], Turkey [25], Spain [26], Argentina [27], Germany [28], Portugal [29], and Italy [30]. Based on the 36-item CERQ, a 18-item short version was validated by Garnefski & Kraaij [15] in a sample of 611 adults aged 18–65 years in the Netherlands, maintaining the original nine-factor model and demonstrating satisfactory internal consistency and criterion validity. This short 18-item version has been validated in Turkey and in Peru, respectively in a sample of 317 undergraduate university students [31] and in a sample of 286 students [32]. In 2018, the psychometric properties of a 18-item version and a 27-item version of the scale have been explored also by Holgado-Tello and colleagues [33] and a 18-item version for children has been demonstrated to be a valid and reliable tool for assessing these cognitive emotion regulation strategies during the middle childhood developmental period by Orgilès and co-workers [34].

Both scales (the long and the short version) consist of nine subscales: self-blame, other-blame, rumination, catastrophizing, putting into perspective, positive refocusing, positive reappraisal, acceptance, and planning. The first four strategies are considered maladaptive strategies because they have been shown to be directly related to depression and anxiety, while the rest are considered adaptive strategies because they may act to protect against these disorders [26,35,36,37].

Although the long original 36-item version has demonstrated good psychometric properties across countries and different populations, in Italy two studies tested its factorial structure yielding inconsistent results. Firstly, Presaghi and Ercolani [30] translated the scale and confirmed its factorial structure, reporting a good fit as in the original version by Garnefski and colleagues [7]. Later, a more recent study by Balzarotti and colleagues [38] did not confirm this result and proposed an alternative abbreviation of the tool into 27 items but maintaining the 9-factor structure. Therefore, testing the psychometric properties of the original version of CERQ (36 items) and adapting the 18-item version, which is not yet available in Italy (although it has proven to be valid in many countries), is a priority. Having a short version of this tool may be useful since the 36-item CERQ may be too long to be administered in certain community, clinical, and research settings, especially when time or resources are limited, as suggested by Orgilès et al. [34]. These authors evidenced that abbreviated versions of questionnaires are recommended for at least three reasons: (1) eliminating repetitious items, (2) avoiding the boredom of responding to similar items, and (3) causing less fatigue in participants.

Furthermore, compared to other instruments assessing emotion regulation, such as the Negative Mood Regulation [39], the Emotion Regulation Questionnaire (ERQ) [4], the Difficulties in Emotion Regulation Scale (DERS) [40], and the Positive and Negative Affect Schedule (PANAS) [41], the CERQ has the advantage to measure a broad range of emotion regulation strategies, focusing purely on cognitive strategies.

The purpose of the present study is to adapt to Italian the short version of the CERQ (CERQ-IS) and test its psychometric properties in two Italian samples of young adults and adolescents. More specifically, the study aimed to test in a group of young adults: (1) the factorial structure of the CERQ-IS by replicating the original nine-factor model with reflective indicators found by Garnefski and Kraij [15]; (2) the internal consistency of each subscale; (3) the criterion and the discriminant validity of the scale; (4) the factorial invariance across gender (i.e., male vs. female); and (5) the factorial invariance across age (i.e., young adults vs. adolescents) by including an additional sample of adolescents.

## 2. Method

### 2.1. Participants and Procedure

A sample of 442 young adults (M_age_ = 21.12; SD = 3.69; 69.1% females) completed a battery of self-report questionnaires including the Italian version of the CERQ, the Emotional Regulation Questionnaire (ERQ) and the Multidimensional Perfectionism Scale (MPS). The sample was recruited between October 2019 and February 2020 among the Student Community of Sapienza University of Rome on a voluntary basis. All participants were informed and invited to participate in a study about emotion regulation and psychological well-being during their lecture time. Among these students, 89% were undergraduates (most of them attended a Bachelor’s in Psychology, and only 6% attended Social Services Bachelor); while 11% were graduate students in Psychology. They were asked to sign a written informed consent and completed the questionnaires anonymously.

With the aim to perform factorial invariance tests across age groups, an additional sample composed of 256 adolescents (M_age_ = 14.81; SD = 0.59; 55.5% males) was recruited between October and December 2019. Adolescents were recruited on a voluntary basis from two public high schools in the urban area of Rome (Italy) through a ‘convenience’ sampling method. Two schools were contacted by telephone and after a detailed explanation of the study was outlined at the first appointment, principals at the two high schools gave their permission to conduct the project. The official study presentation was followed by the distribution of parental consent forms and student consent forms to students two weeks prior to the scheduled data collection. There were no restrictions on participation. After permission from both parents and students was obtained, the paper-and-pencil administration of the questionnaires was performed in 12 classes (grades 9–11). Namely, 21.2% of the respondents attended the first year of high school, 78.4% attended the second year of high school, and 0.4% attended the third year of high school. Participants received no compensation to participate in the study. The assessment sessions lasted about 30–40 min.

The study was approved by the Institutional Review Board of the Department of Psychology at Sapienza University of Rome (Prot. n. 0000149).

### 2.2. Measures

The Cognitive Emotion Regulation Questionnaire (CERQ) [7] was administered in the Italian version as adapted by Presaghi and Ercolani [30]. The original version of the CERQ consists of 36 items divided into nine factors as described below. The 18-item short version validated by Garnefski and Kraaij [15] maintained the original nine-factor model and demonstrated satisfactory internal consistency (alphas range from 0.67 to 0.81) and criterion validity. The main difference between the long and the short version is the reduction of the number of items per scale from four to two. Respondents must indicate how often they have used each of these strategies after having experienced stressful events on a 5-point Likert scale ranging from 1 (almost never) to 5 (almost always). The higher the score, the greater the use of that specific emotion regulation strategy. The scale was intended to yield subscale scores on nine strategies: self-blame, other-blame, rumination, catastrophizing, putting into perspective, positive refocusing, positive reappraisal, acceptance, and planning [15]. For the purpose of testing the psychometric properties of the Italian adaptation of the CERQ short version, and comparing it with the long version, the 36-item version was administered, and the 18-item version was embedded in the longer version.

The Emotion Regulation Questionnaire (ERQ) [4] is a 10-item self-report questionnaire designed to measure respondents’ tendency to use two different emotion regulation strategies: Cognitive Reappraisal (6 items such as “I control my emotions by changing the way I think about the situation I’m in”) and Expressive Suppression (4 items such as “I control my emotions by not expressing them”). Responses are rated on a 7-point Likert scale ranging from 1 (strongly disagree) to 7 (strongly agree), with higher scores indicating higher usage of that strategy. For this study, the Italian version of the ERQ was administered, as validated by Balzarotti and colleagues [42], whose findings confirmed the reliability, factor structure, and validity of the Italian adaptation. In addition, the 2-month test–retest reliability provided evidence for the temporal stability of the Italian ERQ comparable to that of the original version [42]. The omega coefficients in the present sample were 0.859 and 0.747 for Cognitive Reappraisal and Expressive Suppression, respectively.

The Multidimensional Perfectionism Scale—short version (MPS-S) [43] was employed to assess perfectionistic traits. The MPS-S consists of 15 items with a seven-point answering scale ranging from Strongly disagree (1) to Strongly agree (7), with higher scores indicating greater perfectionism. The items reflect three dimensions: self-oriented perfectionism (SOP; e.g., “One of my goals is to be perfect in everything I do”), socially prescribed perfectionism (SPP; e.g., “The better I do, the better I am expected to do”) and other-oriented perfectionism (OOP; e.g., “I have high expectations for the people who are important to me”). Higher scores reflect greater self-oriented, other-oriented, and socially prescribed perfectionism. This version was validated for use in Italy [44] showing acceptable composite reliability coefficients for each of the three sub-scales. The omega coefficients in the present sample were 0.906 for SOP, 0.827 for SPP, and 0.770 for OOP.

### 2.3. Data Analytic Strategy

Data were analysed using IBM SPSS v. 23 (IBM Corporation, Armonk, NY, USA) and Mplus version 8.6 [45].

#### 2.3.1. Preliminary Assumptions

As a first step, skewness and kurtosis values were calculated to explore items’ departure from the univariate normal distribution, where values greater than |1| are considered indicative of non-negligible violations [46]. Additionally, Mardia’s multivariate skewness and kurtosis coefficients were employed to evaluate whether data followed a multivariate normal distribution [47]. Moreover, Little’s missing completely at random (MCAR) test was performed to verify the randomness of the observed missing data [48]. Lastly, the possible influence of common method bias (i.e., the variance that is ascribable to the measurement method rather than to the constructs) was addressed by implementing Harman’s single-factor test on the whole set of CERQ items, e.g., [49].

#### 2.3.2. Factor Structure

With the aim to confirm the original CERQ-IS latent structure found by Garnefski and Kraaij [15], a confirmatory factor analysis (CFA) was carried out positing a nine-factor model: (1) Self-blame; (2) Acceptance; (3) Rumination; (4) Positive Refocusing; (5) Refocus on Planning; (6) Positive Reappraisal; (7) Putting into Perspective; (8) Catastrophising; and (9) Other-blame. For comparison purposes, a CFA was also conducted based on the original CERQ structure (i.e., the full 36-item version) [7]. The latent variables were scaled through the marker-variable method by fixing the loadings of the first indicators on each factor to 1. Following a multifaceted approach to the assessment of model fit [50], several goodness-of-fit indices were considered: the root mean square error of approximation (RMSEA; values below 0.08 indicate a moderate fit) [51], the comparative fit index and the Tucker–Lewis index (CFI and TLI, respectively; values above 0.90 indicate an acceptable fit) [52]; and the standardized root mean squared residual (SRMR; values below 0.08 indicate a good fit to the data) [52]. Chi-square test statistics were reported but not considered in evaluating model fit due to its well-known dependence on sample size [53].

#### 2.3.3. Reliability Analyses

Composite reliability coefficients, as suggested by Fornell & Larcker [54], see also [55], were computed to examine the internal consistency of each CERQ-IS subscale, representing the extent to which a set of items reliably measures the underlying factor. A model-based internal consistency coefficient was preferred to traditional Cronbach’s alpha since the former is based on a highly restricted and often unrealistic psychometric model (alpha requires essentially tau-equivalence) [56]. Composite reliability values of >.70 are considered acceptable in non-exploratory research [56]. Moreover, the reliability of individual items was calculated, which represents the variance of the observed indicator explained by the underlying latent variable [54,55]. Since common guidelines consider standardized factor loadings above 0.50 as satisfactory [56], item reliability was expected to be at least 0.25. As previously, reliability of the full 36-item version was estimated for the sake of comparison.

#### 2.3.4. Validity Analyses

Criterion and discriminant validity were gathered via the analysis of the zero-order correlations with the emotional regulation questionnaire (ERQ) and the Multidimensional perfectionism scale (MPS-S), respectively. Cohen suggested *r* values of 0.10, 0.30, and 0.50 to demarcate small, medium, and large effects, respectively [57].

#### 2.3.5. Factorial Invariance

Factorial invariances tests were performed to determine whether the interpretation of CERQ-IS dimensions was conceptually similar across gender (i.e., males vs. females) and age (i.e., adolescents vs. young adults). With the aim of evaluating subgroup latent factor mean differences, three increasing levels of factorial invariance were tested by means of multiple group confirmatory factor analysis (MG-CFA) following Meredith’s [58] stepwise framework: configural, metric, and scalar invariance. Configural invariance analysis was performed to test whether the latent constructs had the same pattern of factor loadings in each group. Metric invariance was examined by constraining each factor loading to be invariant across the groups. Similarly, constraints on item intercepts were introduced to test for scalar invariance. Considering the sensibility of chi-square tests to trivial deviations from a perfect model in large samples [53], differences in CFI and RMSEA were calculated to compare these nested models and to evaluate the tenability of the imposed constraints. Namely, a criterion of ΔCFI < 0.010 and ΔRMSEA < 0.015 was adopted as supporting the equality constraints [53,59]. Once the scalar invariance was established, comparisons of groups latent means were performed, and results were reported in terms of latent Cohen’s D, see [60].

#### 2.3.6. Sample Size Calculation

Lastly, a post hoc power analysis was conducted to assess the goodness-of-fit upon RMSEA using Kim’s [61] approach. The required sample size (*df* = 99; α = 0.05; Power = 0.80) for the less parsimonious model was 165, confirming the feasibility of further analyses. Moreover, at least 10 participants for each item of the scale were guaranteed according to common recommendations for testing dimensionality and internal consistency, e.g., [62].

## 3. Results

### 3.1. Preliminary Analyses

Items’ statistics of the CERQ-IS are reported in Supplementary Table S1. Specifically, items’ skewness ranged from −0.95 to 1.69, while kurtosis values ranged from −0.88 to 3.34. Mardia’s multivariate skewness and kurtosis coefficients indicated that the data did not follow a multivariate normal distribution (*ps* < 0.001). Accordingly, a robust estimation method was adopted for further factor structure analyses (see Section 3.2). Moreover, missing rates on each item ranged from 0.7% to 0.9%, and Little’s MCAR test revealed that data were missing completely at random, χ^2^ (169) = 196.87, *p* > 0.05. These results supported the implementation of the Full Information Maximum Likelihood (FIML) approach for handling missing data [63], which use all available data points without listwise deletion and provides unbiased parameter estimates under ignorable missing data conditions [64]. Lastly, Harman’s single-factor test conducted via exploratory factor analysis (principal axis factoring estimation) accounted for 18% of the variance, denying significant issues of common method variance [49]. Consistently, the single-factor model tested through CFA revealed a very poor fit to the data: MLRχ^2^(108) = 5334.128, *p* < 0.001; CFI = 0.306; TLI = 0.264; SRMR = 0.139; RMSEA = 0.134 (90% CI 0.131 to 0.138). Therefore, the data were considered to be free from common method bias.

### 3.2. Confirmatory Factor Analyses

A confirmatory factor analysis (CFA) was conducted to test the nine-factor short model [15]. Due to the violation of the multivariate normality assumption, the robust maximum likelihood (MLR) estimation method was employed; MLR provides maximum likelihood parameter estimates with standard errors and chi-square test statistics that are robust to non-normality [45]. For comparison purposes, another CFA was simultaneously carried out based on the full CERQ structure [7]. With respect to the short version, all goodness-of-fit indices suggested an excellent fit to the data: MLRχ^2^(99) = 136.733, *p* < 0.01; CFI = 0.982; TLI = 0.975; SRMR = 0.030; RMSEA = 0.029 (90% CI 0.016 to 0.041). In contrast, the full 36-item structure showed an unsatisfactory fit to the data: MLRχ^2^(558) = 1266.757, *p* < 0.001; CFI = 0.896; TLI = 0.883; SRMR = 0.076; RMSEA = 0.054 (90% CI 0.050 to 0.058). Table 1 summarises both factorial solutions. Specifically, the CERQ-IS standardized factor loadings ranged from 0.64 to 0.93 (*ps* < 0.001), attesting to a substantial proportion of common variance among the items. Conversely, relatively unsatisfactory weights (<0.50) were observed for the full 36-item version [56]. Inter-correlations among latent factors are reported in Supplementary Table S2.

### 3.3. Reliability and Validity

As shown in Table 2, the composite reliability coefficients of the CERQ-IS subscales ranged from 0.66 to 0.88. Except for rumination that was minimally acceptable (0.66), five subscales had an acceptable (>0.70) and three had a good (>0.80) reliability, meaning that the scale can be considered to yield reliable scores in each factor. Item reliability indices were satisfactory, ranging between 0.41 to 0.87. Additionally, composite and item reliability indicators of the full CERQ scale were estimated for the sake of comparison (see Table 2 and Table 3).

Criterion validity, which according to the APA dictionary indicates “how well a test correlates with an established standard of comparison” [65], was analysed by calculating zero-order correlations between the CERQ-IS and the ERQ subscales. Namely, the reappraisal score of ERQ was moderately and positively associated with adaptive emotion regulation strategies such as refocusing on planning, putting into perspective, and positive reappraisal (*ps* < 0.001). Small positive associations were observed between the reappraisal score of ERQ and the acceptance and positive refocusing dimensions (*ps* < 0.001), whilst small negative associations were found between the reappraisal score of ERQ and catastrophizing and other-blame dimensions (*ps* < 0.01). Conversely, the ERQ suppression score was positively associated with other-blame and negatively with positive reappraisal subscales of the CERQ-IS (*ps* < 0.01).

Moreover, discriminant validity, defined as “the extent to which measures of theoretically distinct constructs are unrelated empirically to one another” [66] (p. 82) was explored by calculating zero-order correlations between the CERQ-IS and the MPS-S. Namely, the discriminant validity of the scale was confirmed by non-significant or low correlations with the multidimensional perfectionism facets (MPS-S subscales) except for the relationship between other-blame and OOP (r = 0.327, *p* < 0.001). Validity analyses are summarised in Table 3.

Lastly, the degree of overlap between the CERQ versions was estimated by computing zero-order correlations between the same CERQ-IS and full CERQ subscales. These coefficients ranged from 0.812 to 0.952, which constitute between 66% and 91% of variance shared between the long and short version subscales (see Supplementary Table S3).

### 3.4. Tests of Factorial Invariance across Gender and Age

First, factorial invariance tests across gender were implemented (males n = 136; females n = 304). The model fitted well when tested separately on males and females, as well as when tested simultaneously on the two groups, confirming the tenability of the configural invariance model: MLRχ^2^(198) = 258.225, *p* < 0.01; CFI = 0.975; TLI = 0.961; SRMR = 0.043; RMSEA = 0.037 (90% CI 0.023 to 0.049). Next, constraints on factor loadings were introduced and metric invariance was established (ΔCFI = 0; ΔRMSEA = 0). Finally, the difference between the metric and scalar invariance models was negligible (ΔCFI = 0.005; ΔRMSEA = 0.002). Parameter estimates of the scalar invariance model are reported in Supplementary Table S4. After demonstrating that the scalar invariance model was tenable, latent means differences across gender were evaluated. Means values were fixed to 0 in the group of males (i.e., reference group) and freely estimated in the group of females. Compared to males, females showed higher latent means on self-blame (Cohen’s D = 0.34, *p* < 0.05) and putting into perspective (Cohen’s D = 0.32, *p* < 0.01) dimensions. Contrarily, females showed a lower latent mean on the acceptance factor (Cohen’s D = −0.30, *p* < 0.01). No other significant differences were found (*ps* > 0.05).

Subsequently, factorial invariance tests across age were computed by including an additional sample of adolescents (adolescents n = 256; young adults n = 442). First, the model showed a satisfactory fit to the data when tested separately on the adolescents and young adults, as well as when evaluated simultaneously on both groups: MLRχ^2^(198) = 275.417, *p* < 0.001; CFI = 0.977; TLI = 0.965; SRMR = 0.038; RMSEA = 0.033 (90% CI 0.023 to 0.043). By adding equality constraints on factor loadings, metric invariance was established (both CFI and RMSEA values improved). Finally, constraints on item intercepts were introduced showing a meaningless difference between the metric and scalar invariance models (ΔCFI = 0.009; ΔRMSEA = 0.005). Parameter estimates of the scalar invariance model are reported in Supplementary Table S5. As a further step, latent means differences across age were evaluated. Means values were fixed to 0 in the group of adolescents (i.e., reference group) and freely estimated in the group of young adults. Compared to adolescents, young adults reported higher latent means on acceptance (Cohen’s D = 0.42, *p* < 0.001), focus on thought/rumination (Cohen’s D = 0.36, *p* < 0.001), refocus on planning (Cohen’s D = 0.31, *p* < 0.01) and positive reappraisal (Cohen’s D = 0.45, *p* < 0.001) dimensions. On the other hand, young adults showed a lower latent mean on the positive refocusing factor compared to adolescents (Cohen’s D = −0.52. *p* < 0.001). No other significant differences were found (*ps* > 0.05).

Factorial invariance results are summarised in Table 4.

## 4. Discussion

The main objective of this study was to evaluate the psychometric properties of an 18-item short form version of the CERQ for use in research and practice contexts where the regular 36-item version is too long. This version of the scale was evaluated for its factor structure, internal consistency, age and gender measurement equivalence, and criterion and discriminant validity in relation to measures of emotion regulation and perfectionism, respectively. Results suggest that the CERQ-IS is a substantially leaner yet high-performing assessment tool compared to its 36-item version. First, the robustness of the measurement model was supportive of the multidimensional conceptualization of cognitive emotion regulation strategies. In comparison with the full measure, the CERQ-IS showed a fit to the data that was significantly enhanced, particularly with respect to dimensional characteristics, meaning that the model predictions and the data set did not differ significantly [56].

This finding not only indicated that the CERQ-IS is a viable alternative to the original version, but also strongly encouraged the use of the reduced form over the long form of the CERQ for research in time-restricted conditions. However, when reliability was examined, composite reliability coefficients of the long version provided higher values in most of the subscales as compared to the short version. Despite the reduction in the number of items, the internal consistency of the CERQ-IS dimensions still proved to be satisfactory to good except for Rumination (i.e., composite reliability = 0.659). This estimate is notably lower than what was found by Garnefski and Kraaij [15], who reported an alpha coefficient of 0.79. This result is in line with previous studies, e.g., [22,67] and suggests that the uncertainty of this subscale may be a reflection of the grouping of items with low factorial weights in the same dimension. Alternatively, the lower reliability of Rumination could be attributed to the nature of the questions themselves. Some authors suggest that one of the items on the Rumination factor, “I often think about how I feel about what I have experienced”, may capture something resembling tendencies for speculation rather than rumination [68].

Criterion and discriminant validity of the CERQ-IS was established using Pearson’s product-moment correlation coefficients between CERQ-IS subscales and closely related constructs of the ERQ, as well as the MPS-S, respectively. The choice of the ERQ for criterion validity was based on the need for a well-validated instrument capturing emotion regulation facets [69]. Results indicated small to moderate significant and positive associations between CERQ-IS adaptive subscales and ERQ-reappraisal, and small significant positive and negative correlations of ERQ-suppression with Other-blame and Positive reappraisal, respectively. The moderate association between CERQ-IS Positive reappraisal and ERQ-Reappraisal reflects the similar construct measured, namely reframing negative events by focusing on their positive features [70], although CERQ-IS Positive reappraisal is less emotion-focused than the Reappraisal subscale of the ERQ [71]. Furthermore, results indicated moderate significant and positive associations between ERQ-Reappraisal and CERQ-IS Putting into perspective and Refocus on planning. These findings support previous evidence [72] and highlight the importance of habitual antecedent-focused reinterpretation of stressful events in these CERQ strategies. Finally, small significant and positive correlations of Reappraisal with Acceptance and Positive refocusing were observed, suggesting that the reinterpretation of an event could also implicate awareness of the impossibility of changing unpleasant aspects of an event as well as directing attention to pleasant thoughts that diminish the effect of the stressful situation [73].

The finding that ERQ-Reappraisal was positively related to functional CERQ-IS strategies replicates previous findings, e.g., [70,72] and suggests that the attempt to reinterpret the meaning of a situation in order to modify its emotional impact is related to a higher tendency to activate adaptive emotional cognitive responses. Traditionally, reappraisal has been associated with protective mental health factors, with some scholars stating that this strategy could promote efficient coping and reduce the risk of mental disorder onset [74]. Conversely, ERQ-Suppression was merely associated with two CERQ-IS dimensions, namely Other-blame and Positive reappraisal. This finding may be explained by the fact that CERQ (either the long and the short version) does not explore the strategy of suppression [7,15] and thus may show negligible to low correlations with this ERQ factor. Future studies should evaluate the criterion validity of the CERQ-IS using other emotion regulation instruments, such as the DERS [40] which captures a broad range of emotion regulation facets closely linked to the CERQ-IS (e.g., non-acceptance).

However, the positive and negative associations of ERQ-Suppression with CERQ-IS Other-blame and Positive reappraisal, respectively may be explained in several ways. The characteristics of suppression to inhibit or reduce ongoing emotion-expressive behavior [75] could also result in a tendency to attribute one’s undesirable emotional state to external contexts or people (e.g., external attribution bias) [76]. Therefore, it can be speculated that the tendency to blame other people for negative emotional events may be related to the difficulty to express them. As regards the negative association found between ERQ-Suppression and Positive Reappraisal, it could reflect the opposite response-focused vs. antecedent-focused nature of these two forms of emotion regulation [2]. Based on results from discriminant validity calculations, non-significant or low correlations between multidimensional perfectionism facets and CERQ-IS subscales were observed. One exception was OOP which showed a moderate association with the other-blame dimension. This result expanded previous findings on external attribution of blame to others [77] and provided additional support to the evidence that individuals with other-directed behavior may have different sanctioning styles such as blaming others for misfortunes [78].

The last objective of the present study was to examine measurement invariance and latent mean differences of CERQ-IS scores across two samples of young adults and adolescents. Regards measurement invariance, configural, metric, and scalar invariances of CERQ-IS across the two samples were established. Configural invariance suggests that the model fits the data reasonably well in both groups, thus indicating a common nine-factor structure shared between the samples. This result is relevant insofar as it supports the utility of a multidimensional approach to evaluate specific aspects of emotion regulation in adolescent functioning. Although it is generally argued that emotional and cognitive abilities are being mastered during adolescence [79], the present results apparently suggest that the same multidimensional differentiation of emotion regulation strategies traditionally employed among adults is also applicable to adolescents, as previous studies recommended [14].

Since scalar measurement invariance of CERQ-IS across young adults and adolescents was achieved, the latent means of CERQ-IS dimensions were compared between these groups. Findings suggested that young adults tended to utilize more Acceptance, Rumination, Refocus on planning, and Positive reappraisal than adolescents with moderate differences observed for each of these strategies. Overall, these results concur with previous studies evidencing that adults reported higher use of adaptive CERQ strategies than adolescents [79]. Consistently, the refinement of cognitive emotion regulation strategies may depend on the number of emotion-eliciting stressful occasions which usually grows as the individual grows older [79]. The higher use of acceptance detected among young adults may reflect the complex nature of this CERQ strategy, as it could involve mindfulness components that become effective and stable with advancing age [73]. The greater tendency of young adults in planning how to face negative events (i.e., Refocus on planning) could be explained by the fact that adolescents often are unprepared to effectively cope with new developmental challenges [80]. Moreover, the difference concerning Positive reappraisal may suggest that adolescents are less likely to reframe unpleasant life events in terms of personal growth [79].

Results concerning Rumination somewhat contradicted previous research finding that dysfunctional rumination was more often reported during middle and late adolescence than during emerging adulthood [81]. However, it is plausible that the proneness of negative repetitive thought is particularly prevalent in young adulthood, as during this challenging transitional period of life individuals appear to employ this strategy to face negative events [82]. Finally, the evidence that adolescents from the current study presented greater engagement in positive refocusing than young adults substantiated previous findings in the literature [79]. One may argue that, since adolescents often experience difficulties in coping with their emotions [83], they could engage in focusing on other pleasant events as an attempt to escape from the awareness of negative internal states.

The findings in support of gender measurement invariance for ratings of the CERQ-IS indicated that this scale measures conceptually similar constructs across males and females and that differences in latent means were not confounded by systematic discrepancies in responses given by these two groups. It was observed that compared to males, females showed higher levels of self-blame and lower scores on acceptance. These results substantiated previous findings in the literature [84,85,86] and would seem to suggest that females could tend to rely on emotion-focused coping strategies to a greater extent than men [36]. One potential explanation for this finding could be the higher tendency of females to be attentive to their moods which may hinder access to positive cognitions and thus result in maladaptive coping strategies [87]. However, female participants in the present sample also reported higher scores on putting into perspective dimension, which is an adaptive CERQ strategy, as compared to males. This result is similar to previous research [86] and highlighted the need to investigate mechanisms underlying gender differences in the dispositional use of cognitive strategies.

The current study should be considered in light of the following limitations. First, its cross-sectional nature precludes causal inferences of the relationships among the variables explored, as well as the possibility to establish a test re-test reliability of the CERQ-IS. Further analyses are needed to additionally evaluate the stability of the measurements over time. Second, the lack of a measure of psychopathological symptoms did not allow verification of the criterion validity with other constructs relevant to mental health. Third, although the ERQ [4] is one of the best validated emotion regulation measures, it is restricted to capturing only two facets of emotion regulation (i.e., suppression, reappraisal) and the inclusion of other reliable instruments assessing multifaceted emotion regulation aspects should be considered in future investigations. Fourth, given the small sample size and typology, the findings might not be transferable to other populations (e.g., clinical). Fifth, as the CERQ-IS was administered embedded in the longer 36-item version, replication of the present study results with an independently administered CERQ-IS is called for. Moreover, as context plays a central role in ER processes [88], it fruitful avenue for further research is to assess emotion flexibility, conceived as the ability to adopt different strategies in response to changing contextual demands and stressful events [89], as it has been assumed that the effectiveness of different ER strategies is dependent on the specific situation (e.g., exam-related contexts in student populations) [90].

One last limitation of this study is inherent to the evaluation of the psychometric properties of the CERQ-IS from items embedded in the original version of the scale, rather than a stand-alone short form, which suggests that CERQ-IS items may function in a different way if applied alone [91].

Despite the aforementioned limitations, the current study contributes to the literature on emotion regulation strategies by promoting the use of the CERQ-IS in research contexts especially when time or resources are limited. Moreover, this work supported previous evidence on the measurement invariance of adolescent CERQ dimensions over time [92] thus encouraging the employment of this tool also among youngsters. Moreover, the employment of a short version of the scale could facilitate clinical practices and the implementation of effective interventions aimed at the reduction of maladaptive emotion regulation and psychological difficulties. Clinicians should consider that the same multidimensional differentiation of CERQ strategies traditionally employed among young adults is also applicable to adolescents. This suggests that theoretical models designed for the prediction of psychopathology in these populations could be implemented by considering a parallel differentiation of emotion regulation strategies. These topics are reserved for future work.

## Figures and Tables

**Table 1:** Factor structure of the 18-item short (CERQ-IS) and 36-item full (Full CERQ) versions of the CERQ. All the factor loadings obtained from the CFA solutions are reported in completely standardized metric and are statistically significant (*p* < 0.001). Abbreviations: CERQ, cognitive emotion regulation questionnaire; CERQ-IS, Italian short version of the cognitive emotion regulation questionnaire; IRI, item reliability index.

Subscales and Items	CERQ-IS		Full CERQ	
	Factor Loading	IRI	Factor Loading	IRI
Self-blame				
I feel that I am the one to blame for it			0.575	0.331
I feel that I am the one who is responsible for what has happened	0.639	0.409	0.751	0.563
I think about the mistakes I have made in this matter			0.624	0.390
I think that basically the cause must lie within myself	0.866	0.751	0.703	0.494
Acceptance				
I think that I have to accept that this has happened	0.935	0.874	0.818	0.670
I think that I have to accept the situation	0.835	0.697	0.955	0.912
I think that I cannot change anything about it			0.182	0.033
I think that I must learn to live with it			0.428	0.183
Rumination				
I often think about how I feel about what I have experienced	0.704	0.496	0.644	0.415
I am preoccupied with what I think and feel about what I have experienced	0.698	0.488	0.728	0.530
I want to understand why I feel the way I do about what I have experienced			0.694	0.482
I dwell upon the feelings the situation has evoked in me			0.748	0.559
Positive refocusing				
I think of nicer things than what I have experienced			0.730	0.532
I think of pleasant things that have nothing to do with it	0.789	0.622	0.805	0.648
I think of something nice instead of what has happened	0.883	0.780	0.867	0.751
I think about pleasant experiences			0.845	0.714
Refocus on planning				
I think of what I can do best			0.868	0.753
I think about how I can best cope with the situation			0.878	0.771
I think about how to change the situation	0.762	0.580	0.727	0.529
I think about a plan of what I can do best	0.787	0.619	0.737	0.543
Positive reappraisal				
I think I can learn something from the situation	0.780	0.609	0.623	0.388
I think that I can become a stronger person as a result of what has happened	0.737	0.543	0.621	0.385
I think that the situation also has its positive sides			0.929	0.864
I look for the positive sides to the matter			0.887	0.787
Putting into perspective				
I think that it all could have been much worse			0.705	0.498
I think that other people go through much worse experiences			0.857	0.735
I think that it hasn’t been too bad compared to other things	0.810	0.656	0.806	0.649
I tell myself that there are worse things in life	0.867	0.752	0.867	0.751
Catastrophizing				
I often think that what I have experienced is much worse than what others have experienced			0.362	0.131
I keep thinking about how terrible it is what I have experienced	0.877	0.770	0.882	0.778
I often think that what I have experienced is the worst that can happen to a person			0.517	0.267
I continually think how horrible the situation has been	0.857	0.735	0.854	0.729
Other-blame				
I feel that others are to blame for it			0.664	0.440
I feel that others are responsible for what has happened	0.885	0.784	0.897	0.804
I think about the mistakes others have made in this matter			0.735	0.540
I feel that basically the cause lies with others	0.667	0.444	0.655	0.429

**Table 2:** Descriptive statistics for CERQ-IS subscales, and composite reliability coefficients based on both short and full versions of the CERQ for the sake of comparison. Abbreviations: CERQ, cognitive emotion regulation questionnaire; CERQ-IS, Italian short version of the cognitive emotion regulation questionnaire; SD, standard deviation.

Subscale	Mean ± SD (Short Version, CERQ-IS)	Composite Reliability (Short Version, CERQ-IS)	Composite Reliability (Original Version, Full CERQ)
Self-blame	4.86 (1.78)	0.729	0.760
Acceptance	6.91 (1.91)	0.880	0.720
Rumination	6.75 (1.94)	0.659	0.797
Positive refocusing	4.41 (1.91)	0.824	0.886
Refocus on planning	7.46 (1.72)	0.750	0.880
Positive reappraisal	8.03 (1.79)	0.731	0.856
Putting into perspective	6.16 (2.12)	0.826	0.884
Catastrophizing	4.49 (2.04)	0.858	0.765
Other-blame	3.77 (1.57)	0.757	0.830

**Table 3:** Pearson’s correlations between CERQ-IS subscales and ERQ reappraisal and suppression scores, and MPS-S subscales scores. ** *p* < 0.01; * *p* < 0.05. Abbreviations: CERQ-IS, Italian short version of the cognitive emotion regulation questionnaire; ERQ, emotion regulation questionnaire; MPS-S, Multidimensional Perfectionism Scale—short version.

CERQ-IS Subscales	ERQ_ Reappraisal	ERQ_ Suppression	MPS-S_Self-Oriented Perfectionism	MPS-S_Other-Oriented Perfectionism	MPS-S_Socially Prescribed Perfectionism
Self-blame	−0.079	0.020	0.147 **	0.054	0.220 **
2. Acceptance	0.271 **	0.017	−0.089	0.007	−0.065
3. Rumination	−0.041	0.052	0.197 **	0.077	0.175 **
4. Positive refocusing	0.294 **	−0.057	−0.088	0.039	−0.062
5. Refocus on planning	0.321 **	−0.087	0.077	0.060	−0.100
6. Positive reappraisal	0.409 **	−0.135 **	−0.013	0.021	−0.133 **
7. Putting into perspective	0.365 **	−0.066	−0.043	0.035	−0.085
8. Catastrophizing.	−0.214 **	0.068	0.119 *	0.023	0.243 **
9. Other-blame	−0.145 **	0.135 **	0.219 **	0.327 **	0.286 **

**Table 4:** Factorial invariance tests of the CERQ-IS across gender and age. Different scaling correction factors are related to χ^2^ statistics since robust maximum likelihood estimation method (MLR) was implemented.

Gender as Grouping Variable							
Model	MLRχ^2^ (DFs)	RMSEA (90% CI)	ΔRMSEA	CFI	ΔCFI	TLI	SRMR
Males	108.601 (99)	0.027 (0.000–0.054)		0.986		0.979	0.048
Females	147.325 (99)	0.040 (0.026–0.053)		0.971		0.956	0.040
Configural invariance	258.225 (198)	0.037 (0.023–0.049)		0.975		0.961	0.043
Metric invariance	268.299 (207)	0.037 (0.023–0.049)	0	0.975	0	0.962	0.043
Scalar invariance	287.066 (216)	0.039 (0.026–0.050)	0.002	0.970	0.005	0.958	0.045
**Age as grouping variable**							
**Model**	**MLR χ^2^ (DFs)**	**RMSEA (90% CI)**	**ΔRMSEA**	**CFI**	**ΔCFI**	**TLI**	**SRMR**
Adolescents	138.566 (99)	0.040 (0.022–0.054)		0.964		0.944	0.048
Young adults	136.733 (99)	0.029 (0.016–0.041)		0.982		0.975	0.030
Configural invariance	275.417 (198)	0.033 (0.023–0.043)		0.977		0.965	0.038
Metric invariance	281.548 (207)	0.032 (0.022–0.041)	Improved	0.978	Improved	0.968	0.040
Scalar invariance	320.707 (216)	0.037 (0.028–0.046)	0.005	0.969	0.009	0.956	0.043

## Data Availability

The data presented in this study are available upon reasonable request from the corresponding author.

## Supplementary Materials

The following supporting information can be downloaded at: https://www.mdpi.com/article/10.3390/bs12120474/s1, Table S1: Items’ statistics of the CERQ-IS. Table S2: Inter-correlations among latent factors of the CERQ-IS. Table S3: Pearson’s correlations between CERQ-IS and full CERQ subscales. Table S4: Factor loadings of the CERQ-IS obtained from the scalar invariance model (gender as grouping variable). Table S5: Factor loadings of the CERQ-IS obtained from the scalar invariance model (age as grouping variable).
