# Supplementary material for: Cognitive Emotion Regulation Questionnaire—Short: Reliability, Validity, and Measurement Invariance of the Italian Version
Source: Behav Sci (Basel). 2022 Nov 24;12(12):474. doi: 10.3390/bs12120474 (PMC9774580; doi:10.3390/bs12120474)
Supplement: Supplementary file 1 [file behavsci-12-00474-s001.zip › behavsci-2015218-supplementary.pdf]

SUPPLEMENTARY DOCUMENT 1

Subscales and items	Mean	SD	Skewness (SE)	Kurtosis (SE)
Self-blame				
I feel that I am the one who is responsible for what has happened	2.87	1.01	0.06 (0.11)	-0.60 (0.23)
I think that basically the cause must lie within myself	2.00	1.00	0.91 (0.11)	0.34 (0.23)
Acceptance				
I think that I have to accept that this has happened	3.48	0.99	-0.29 (0.11)	-0.42 (0.23)
I think that I have to accept the situation	3.44	1.04	-0.32 (0.11)	-0.48 (0.23)
Rumination				
I often think about how I feel about what I have experienced	3.50	1.12	-0.44 (0.11)	-0.58 (0.23)
I am preoccupied with what I think and feel about what I have experienced	3.28	1.11	-0.29 (0.11)	-0.58 (0.23)
Positive refocusing				
I think of pleasant things that have nothing to do with it	2.28	1.08	0.70 (0.11)	-0.20 (0.23)
I think of something nice instead of what has happened	2.14	0.99	0.74 (0.11)	-0.05 (0.23)
Refocus on planning				
I think about how to change the situation	3.75	0.91	-0.22 (0.11)	-0.65 (0.23)
I think about a plan of what I can do best	3.73	0.98	-0.37 (0.11)	-0.65 (0.23)
Positive reappraisal				
I think I can learn something from the situation	4.06	0.95	-0.95 (0.11)	0.54 (0.23)
I think that I can become a stronger person as a result of what has happened	3.98	1.04	-0.79 (0.11)	-0.12 (0.23)
Putting into perspective				
I think that it hasn't been too bad compared to other things	2.95	1.09	0.04 (0.11)	-0.65 (0.23)
I tell myself that there are worse things in life	3.24	1.20	-0.19 (0.11)	-0.88 (0.23)
Catastrophizing				
I keep thinking about how terrible it is what I have experienced	2.36	1.08	0.50 (0.11)	-0.44 (0.23)
I continually think how horrible the situation has been	2.14	1.09	0.73 (0.11)	-0.29 (0.23)
Other-blame				
I feel that others are responsible for what has happened	2.25	0.98	0.49 (0.11)	-0.32 (0.23)
I feel that basically the cause lies with others	1.53	0.78	1.69 (0.11)	3.34 (0.23)

Table S1. Items' statistics of the CERQ-IS. Abbreviations: CERQ-IS, Italian short version of the cognitive emotion regulation questionnaire; SD, standard deviation; SE standard error.

Latent factor	1	2	3	4	5	6	7	8	9
1. Self-blame									
2. Acceptance	-.130*								
3. Rumination	.425**	.035							
4. Positive refocusing	-.183**	.194**	-.144*						
5. Refocus on planning	-.188**	.221**	.070	.222**					
6. Positive reappraisal	-.187**	.385**	.083	.198**	.602**				
7. Putting into perspective.	-.080	.212**	-.086	.365**	.306**	.401**			
8. Catastrophizing	.431**	-.163**	.505**	-.143*	-.175**	-.228**	-.133		
9. Other-blame	.105	-.067	.211**	.138*	-.059	-.112	.026	.275**	

Table S2. Inter-correlations among latent factors of the CERQ-IS. ** *p* <.01; * *p* <.05. Abbreviations: CERQ-IS, Italian short version of the cognitive emotion regulation questionnaire.

CERQ-IS subscale	Full CERQ subscale								
	1	2	3	4	5	6	7	8	9
1. Self-blame	.895**	.029	.319**	-.195**	-.172**	-.180*	-.079	.301**	.074
2. Acceptance	-.063	.812**	.040	.161**	.239**	.349**	.205**	-.112*	-.042
3. Rumination	.380**	.139**	.895**	-.099*	.018	-.045	-.103*	.299**	.175**
4. Positive refocusing	-.185**	.079	-.097*	.952**	.202**	.245**	.331**	-.043	.131**
5. Refocus on planning	-.122*	-.055	.104*	.228**	.936**	.468**	.255**	-.131**	-.039
6. Positive reappraisal	-.089	.145**	.105*	.217**	.524**	.875**	.328**	-.170**	-.076
7. Putting into perspective	-.078	.108*	-.115*	.358**	.286**	.395**	.940**	-.069	.022
8. Catastrophizing	.349**	.053	.359**	-.181**	-.201**	-.328**	-.147**	.908**	.264**
9. Other-blame	.064	.019	.118*	.108*	-.086	-.096*	-.003	.258**	.917**

Table S3. Pearson's correlations between CERQ-IS and full CERQ subscales. ** *p* <.01; * *p* <.05. Abbreviations: CERQ, cognitive emotion regulation questionnaire; CERQ-IS, Italian short version of the cognitive emotion regulation questionnaire.

Subscales and items	Males	Females
Self-blame		
I feel that I am the one who is responsible for what has happened	.586	.685
I think that basically the cause must lie within myself	.870	.816
Acceptance		
I think that I have to accept that this has happened	.917	.937
I think that I have to accept the situation	.707	.893
Rumination		
I often think about how I feel about what I have experienced	.612	.716
I am preoccupied with what I think and feel about what I have experienced	.623	.735
Positive refocusing		
I think of pleasant things that have nothing to do with it	.886	.782
I think of something nice instead of what has happened	.852	.862
Refocus on planning		
I think about how to change the situation	.712	.797
I think about a plan of what I can do best	.750	.787
Positive reappraisal		
I think I can learn something from the situation	.814	.758
I think that I can become a stronger person as a result of what has happened	.770	.721
Putting into perspective		
I think that it hasn’t been too bad compared to other things	.762	.737
I tell myself that there are worse things in life	.979	.909
Catastrophizing		
I keep thinking about how terrible it is what I have experienced	.864	.899
I continually think how horrible the situation has been	.875	.834
Other-blame		
I feel that others are responsible for what has happened	.815	.825
I feel that basically the cause lies with others	.784	.688

Table S4. Factor loadings of the CERQ-IS obtained from the scalar invariance model (gender as grouping variable). Abbreviations: CERQ-IS, Italian short version of the cognitive emotion regulation questionnaire.

Subscales and items	Adolescents	Young adults
<u>Self-blame</u>		
I feel that I am the one who is responsible for what has happened	0.661	0.623
I think that basically the cause must lie within myself	0.800	0.879
<u>Acceptance</u>		
I think that I have to accept that this has happened	0.905	0.967
I think that I have to accept the situation	0.783	0.805
<u>Rumination</u>		
I often think about how I feel about what I have experienced	0.699	0.716
I am preoccupied with what I think and feel about what I have experienced	0.653	0.686
<u>Positive refocusing</u>		
I think of pleasant things that have nothing to do with it	0.680	0.727
I think of something nice instead of what has happened	0.895	0.949
<u>Refocus on planning</u>		
I think about how to change the situation	0.752	0.756
I think about a plan of what I can do best	0.854	0.792
<u>Positive reappraisal</u>		
I think I can learn something from the situation	0.778	0.822
I think that I can become a stronger person as a result of what has happened	0.690	0.686
<u>Putting into perspective</u>		
I think that it hasn't been too bad compared to other things	0.739	0.835
I tell myself that there are worse things in life	0.744	0.841
<u>Catastrophizing</u>		
I keep thinking about how terrible it is what I have experienced	0.809	0.877
I continually think how horrible the situation has been	0.836	0.858
<u>Other-blame</u>		
I feel that others are responsible for what has happened	0.722	0.892
I feel that basically the cause lies with others	0.490	0.661

Table S5. Factor loadings of the CERQ-IS obtained from the scalar invariance model (age as grouping variable). Abbreviations: CERQ-IS, Italian short version of the cognitive emotion regulation questionnaire.
